# Waist circumference and waist-to-height ratio of Hong Kong Chinese children

**DOI:** 10.1186/1471-2458-8-324

**Published:** 2008-09-22

**Authors:** Rita YT Sung, Hung-Kwan So, Kai-Chow Choi, Edmund AS Nelson, Albert M Li, Jane AT Yin, Charlotte WL Kwok, Pak-Cheung Ng, Tai-Fai Fok

**Affiliations:** 1 Department of Paediatrics, The Chinese University of Hong Kong, Hong Kong Special Administrative Region, the People's Republic of China; 2 Centre for Clinical Trials and Epidemiological Research, The Chinese University of Hong Kong, Hong Kong Special Administrative Region, the People's Republic of China

## Abstract

**Background:**

Central body fat is a better predictor than overall body fat for cardiovascular (CV) risk factors in both adults and children. Waist circumference (WC) has been used as a proxy measure of central body fat. Children at high CV risk may be identified by WC measurements. Waist-to-height ratio (WHTR) has been proposed as an alternative, conveniently age-independent measure of CV risk although WHTR percentiles have not been reported. We aim to provide age- and sex-specific reference values for WC and WHTR in Hong Kong Chinese children.

**Methods:**

Cross sectional study in a large representative sample of 14,842 children aged 6 to 18 years in 2005/6. Sex-specific descriptive statistics for whole-year age groups and smoothed percentile curves of WC and WHTR were derived and presented.

**Results:**

WC increased with age, although less after age 14 years in girls. WHTR decreased with age (particularly up to age 14). WHTR correlated less closely than WC with BMI (r = 0.65, 0.59 cf. 0.93, 0.91, for boys and girls respectively).

**Conclusion:**

Reference values and percentile curves for WC and WHRT of Chinese children and adolescents are provided. Both WC and WHTR are age dependent. Since the use of WHRT does not obviate the need for age-related reference standards, simple WC measurement is a more convenient method for central fat estimation than WHRT.

## Background

Central obesity in both adults [1-3] and children [4-6] is related to cardiovascular (CV) risk factors (hyperlipidemia, insulin resistance and hypertension). Waist circumference (WC) and WC-derived indices such as waist-to-hip ratio and waist-to-height ratio (WHTR) have been used as proxy measures of central obesity, the former being widely used in adults [7,8]. There is growing evidence however that waist circumference (WC) alone correlates more strongly than waist-to-height ratio (WHTR) with CV risk, in adults [2] as well as in children [9-11]. Previous studies have shown that adults from a number of South and East Asian countries are predisposed to central obesity and insulin resistance [12,13]. This ethnic difference of increased body fat distribution in the trunk region has also been observed in adolescents [14] and prepubertal children [15] of mainly East Asian origin, highlighting the importance of ethnic-specific studies.

Standard WC cutoffs for high CV risk have been proposed for adults [16-18] and are widely used, but measurement of WC is not yet common practice in growing children. Age-related percentiles have been reported for children from a number of different countries: Italy [19]; Spain [20]; Cyprus [21]; United Kingdom [22]; Canada [23]; USA [24]; Holland [25]; Australia [26]; and Iran [27] (Table 1), but, to our knowledge, the only published WC data in Chinese children in the English literature is our relatively small study of 6 to 12 year old children [11], drawn from the same population as the present study.

**Table 1:** A summary of population studies on children's waist circumference

Countries	Sample size	Age range	Year of data	Authors/publication year
Italy^a^	F 1418, M 1440	6–14	1993	Zannolli et al., 1996 [19]
Spain^b^	F 659, M 701	6–14.9	1996	Moreno et al., 1999 [20]
Cyprus^a^	F 1214, M 1258	6–17	1999–2000	Savva et al., 2001 [21]
UK^b^	F 3585, M 4770	5–16.9	1988	McCarthy et al., 2001 [22]
Canada^c^	F 1524, M 1540	10.5–18.5	1981	Katzmarzyk, 2004 [23]
USA^d^	F 4944, M 4769	2–18	1988–94	Fernandez et al., 2004 [24]
Netherlands^b^	F 7018, M 7482	0–21	1996–1997	Fredriks et al., 2005 [25]
Australia^a^	F 4162, M 4277	7–15	1985	Eisenmann et al., 2005 [26]
Iran^b^	F 10858, M 10253	6–18	2003–2004	Kelishadi et al., 2007 [27]
Present study^b^	F 7370, M 7472	6–18	2005–2006	

WC measured at ^a ^at umbilical level, ^b ^midway between the lowest rib and the superior border of the iliac crest, ^c ^at the point of noticeable waist narrowing, ^d ^just above the uppermost lateral border of the right ilium

WHTR has been proposed as a convenient alternative measure for assessing central fatness in children, on the basis that it is relatively age-independent and that in normalising for growth it might obviate the need for age-related reference charts [28-30]. "Keep your waist circumference to less than half your height" (i.e. WHTR 0.5) is a seductively simple target [31]. However WHTR percentiles have not been reported and the generality of its independence of ethnic and age differences is questionable.

In 1993 a growth survey of 25,000 Hong Kong children from birth to 18 y was undertaken and growth charts for weight, height, weight-for-height and body mass index were developed [32]. WC was not measured. We now report a further growth study of 14,842 ethnic Chinese children aged 6 to 18 y undertaken in 2005/2006, using similar methods but including WC measurement to provide reference values for WC and WHTR.

## Methods

### Sampling method

A list of all schools in Hong Kong was compiled from data held by the Department of Education. One primary school and one secondary school were selected randomly from each of the 18 Districts in Hong Kong. Two classes in each grade were then selected. (see article on "Secular changes in height, weight and body mass index in Hong Kong Children" for further details). All students of the selected classes were invited to join the study. A fact sheet explaining the purpose and procedure was given to each student and their parents. Seven percent of the primary and 10% of the secondary school students declined to participate. The parents of all participants were invited to complete a questionnaire providing demographic information including gestation, birth weight, feeding pattern and family or personal history of risk factors for obesity. The study was approved by the Joint Chinese University of Hong Kong New Territories East Cluster Clinical Research Ethics Committee and the Ethics Committee of the Department of Health of the Hong Kong Government.

### Measurement of growth parameters

A team of 8 trained research staff visited the selected schools on a pre-arranged date to collect the anthropometric data. All instruments were standardised before the examination and the balances were zero calibrated. Standing height without shoes was measured twice using a Harpenden Stadiometer (Holtain, UK) to the nearest 0.1 cm. Body weight was measured with the lightest clothing to the nearest 0.1 kg by an electronic weighting scale (Tanita BF-522, Japan). WC was measured midway between the lowest rib and the superior border of the iliac crest with an inelastic measuring tape at the end of normal expiration to the nearest 0.1 cm. The intra-class (within-observer) correlation coefficients, based on pairs of replicate measurements made by the same observer on 100 subjects on the same day, were 0.998 for weight and height, 0.997 for WC. All data were checked twice by two staff before data entry and then checked for inconsistencies.

### Statistics

Statistical analyses were performed with SPSS 14.0 (SPSS Inc., Chicago, IL). Inter-correlations between BMI and WC as well as WHTR were assessed using Pearson correlation coefficients. Smoothed percentile curves were constructed for WC and WHTR by LMS method [33]. To compare our data with that of children from different ethnic populations, age- and gender-specific 10^th^, 50^th ^and 90^th ^percentile curves from Britain and Iran were plotted with our data on the same chart. Mean WHTR of boys and girls were compared between the present study and the British survey using Student's t-test. All statistical tests were two-sided. A p-value < 0.05 was considered statistically significant.

## Results

**Figure 1:**
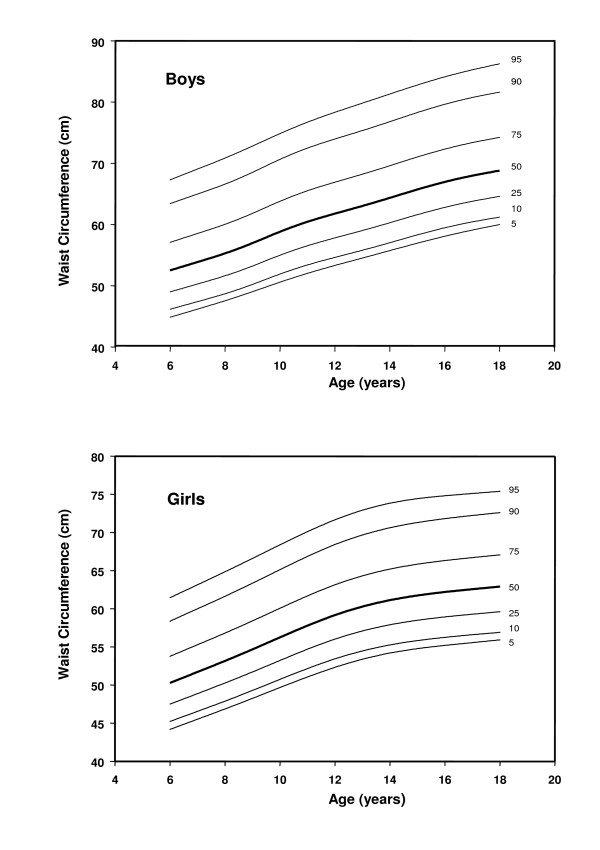
Smoothed percentile curves of waist circumference for Hong Kong Chinese children aged 6 to 18 y.

**Figure 2:**
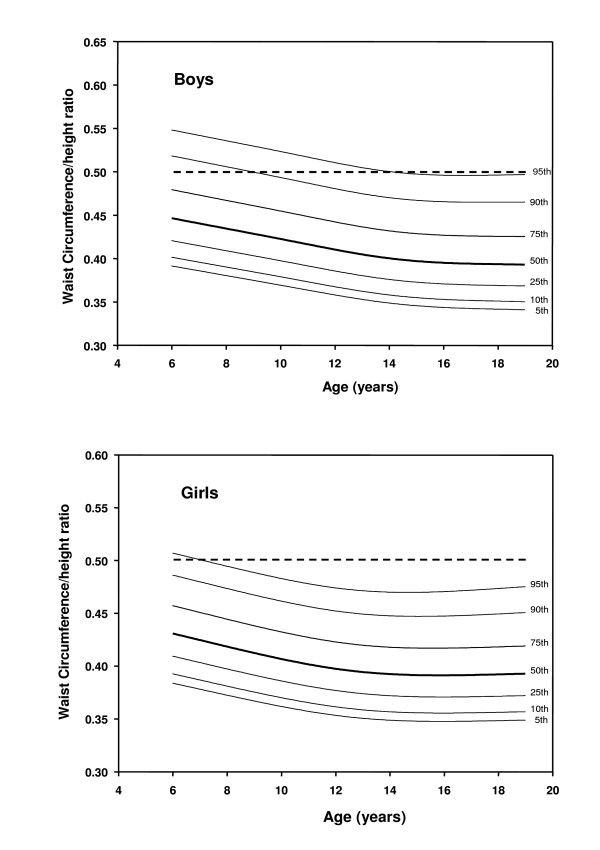
**Smoothed percentile curves of waist circumference to height ratio (WHTR) for Hong Kong Chinese children aged 6 to 18 y.** Dashed line shows superimposed universal cut-off value of 0.5 for all children.

A total of 14,842 children participated in the study (7472 boys and 7370 girls). Sex and age-specific mean weight, height, BMI, WC and WHTR were obtained (Table 2) and then smoothed to develop sex- and age-specific WC and WHTR percentiles (Tables 3 and 4 and Figures 1 and 2). WC was relatively larger in boys than girls and increased with age, though to a smaller extent in girls after age 14 y. WHTR was slightly larger in boys than girls and in both sexes decreased with age but only up to age 14 y and changed little further. Over the age range 14 to 18 y WHTR 0.5 corresponded to the 95^th ^percentile for boys and the 97^th ^percentile for girls (Figure 2). WC correlated more closely than WHTR with BMI (r = 0.93, 0.91 cf. 0.65, 0.59, for boys and girls respectively).

**Table 2:** Sample sizes and mean and standard deviations (SD) for weight, height, BMI, waist circumference (WC) and waist-to-height ratio (WHTR) for Hong Kong Chinese children aged 6 to 18 y

**Sex**	**Age**	**n**	**Weight (kg)**	**Height (cm)**	**BMI**	**WC (cm)**	**WHTR**
**Boys**	6	402	23.9 (4.7)	120.4 (5.5)	16.2 (2.4)	53.7 (5.4)	0.45 (0.04)
(n = 7472)	7	520	26.4 (6.1)	125.7 (5.9)	16.6 (2.7)	55.4 (6.6)	0.44 (0.04)
	8	572	29.3 (6.7)	130.7 (5.9)	17.0 (2.9)	56.9 (6.8)	0.43 (0.05)
	9	629	32.7 (7.8)	135.6 (6.0)	17.6 (3.2)	59.5 (7.8)	0.44 (0.06)
	10	627	37.5 (9.6)	141.2 (6.9)	18.7 (3.7)	62.4 (9.1)	0.44 (0.06)
	11	644	41.8 (10.6)	147.3 (7.9)	19.1 (3.7)	64 0(9.2)	0.43 (0.05)
	12	729	46.6 (11.9)	154.1 (8.6)	19.4 (3.7)	64.6 (9.1)	0.42 (0.05)
	13	657	51.1 (11.3)	161.2 (7.9)	19.6 (3.5)	65.2 (8.2)	0.40 (0.05)
	14	632	54,9 (10.9)	166.3 (6.9)	19.8 (3.4)	66 0(7.7)	0.40 (0.04)
	15	558	59.1 (12.0)	169.8 (5.4)	20.4 (3.8)	68.1 (8.7)	0.40 (0.05)
	16	579	60.6 (11.1)	170.9 (5.8)	20.7 (3.4)	68.8 (8.2)	0.40 (0.05)
	17	553	61.5 (11.0)	171.9 (5.7)	20.8 (3.4)	69.4 (7.8)	0.40 (0.05)
	18	370	62.3 (11.0)	171.7 (5.6)	21.1 (3.3)	70.1 (7.6)	0.41 (0.04)
							
**Girls**	6	377	21.8 (3.8)	118.7 (5.1)	15.4 (1.9)	51.3 (4.2)	0.43 (0.03)
(n = 7370)	7	479	24.9 (5.1)	124.2 (5.5)	16.0 (2.5)	53.3 (5.4)	0.43 (0.04)
	8	504	27.8 (6.0)	129.9 (6.3)	16.3 (2.5)	54.6 (5.8)	0.42 (0.04)
	9	590	30.8 (6.4)	135.1 (6.3)	16.8 (2.6)	56.7 (6.0)	0.42 (0.04)
	10	584	35.3 (8.5)	141.7 (7.0)	17.4 (3.1)	58.5 (7.0)	0.41 (0.04)
	11	599	40.1 (9.3)	148.9 (7.0)	19.0 (3.2)	60.2 (6.9)	0.40 (0.04)
	12	750	44.1 (9.4)	153.1 (6.2)	18.7 (3.3)	61.2 (7.0)	0.40 (0.04)
	13	637	47.8 (8.5)	156.2 (5.4)	19.6 (3.1)	62.2 (6.5)	0.40 (0.04)
	14	656	49.0 (8.6)	157.6 (5.4)	19.7 (3.0)	62.3 (6.2)	0.40 (0.04)
	15	600	50.5 (9.1)	158.3 (5.3)	20.1 (3.3)	63.0 (6.7)	0.40 (0.04)
	16	640	50.7 (8.9)	158.3 (5.3)	20.2 (3.3)	63.2 (6.4)	0.40 (0.04)
	17	568	51.4 (9.2)	158.9 (6.7)	20.3 (3.3)	63.5 (6.3)	0.40 (0.04)
	18	386	51.3 (8.1)	158.6 (5.6)	20.4 (2.9)	63.8 (5.6)	0.40 (0.04)

Age: complete age, e.g. 6 y = 6.00–6.99 y

**Table 3:** Age- and sex-specific waist circumference percentile values (cm) for Hong Kong Chinese children 6 to 18 y of age

			**Percentiles**								
			
**Sex**	**Age**	**n**	**3^rd^**	**5^th^**	**10^th^**	**25^th^**	**50^th^**	**75^th^**	**90^th^**	**95^th^**	**97^th^**
**Boys**	6	402	43.8	44.8	46.2	49.0	52.5	57.1	63.4	67.3	73.0
	7	520	45.0	46.2	47.4	50.3	53.9	58.5	65.0	69.1	74.8
	8	572	46.2	47.5	48.7	51.6	55.3	60.0	66.6	70.9	76.6
	9	629	47.7	49.0	50.2	53.2	57.0	61.8	68.5	72.8	78.7
	10	627	49.3	50.6	51.9	55.0	58.8	63.8	70.6	74.9	81.1
	11	644	50.8	52.0	53.4	56.5	60.4	65.5	72.5	76.7	83.1
	12	729	52.0	53.3	54.6	57.8	61.8	66.9	74.0	78.3	84.7
	13	657	53.1	54.5	55.8	59.0	63.0	68.2	75.3	79.8	86.1
	14	632	54.3	55.7	57.0	60.3	64.3	69.6	76.8	81.3	87.7
	15	558	55.5	56.9	58.3	61.6	65.7	71.0	78.3	82.8	89.3
	16	579	56.7	58.1	59.4	62.8	66.9	72.3	79.6	84.1	90.7
	17	553	57.6	59.1	60.4	63.8	68.0	73.4	80.7	85.3	91.8
	18	370	58.4	60.0	61.2	64.6	68.8	74.2	81.6	86.3	92.7
											
**Girls**	6	377	43.3	44.2	45.2	47.5	50.3	53.8	58.4	61.5	64.9
	7	479	44.6	45.5	46.5	48.9	51.7	55.3	60.0	63.1	66.7
	8	504	45.9	46.9	47.9	50.3	53.2	56.8	61.6	64.8	68.5
	9	590	47.2	48.3	49.3	51.7	54.7	58.4	63.4	66.6	70.4
	10	584	48.6	49.7	50.7	53.2	56.2	60.1	65.1	68.4	72.4
	11	599	50.0	51.1	52.2	54.7	57.8	61.7	66.9	70.1	74.3
	12	750	51.3	52.3	53.4	56.0	59.2	63.1	68.4	71.7	76.0
	13	637	52.3	53.4	54.5	57.1	60.3	64.3	69.7	72.9	77.4
	14	656	53.0	54.2	55.3	57.9	61.1	65.2	70.6	73.8	78.4
	15	600	53.6	54.8	55.8	58.5	61.7	65.8	71.3	74.4	79.2
	16	640	54.0	55.2	56.2	58.9	62.2	66.3	71.8	74.8	79.7
	17	568	54.3	55.6	56.6	59.3	62.6	66.7	72.2	75.1	80.2
	18	386	54.6	55.9	56.9	59.6	62.9	67.1	72.6	75.4	80.6

Age: completed age, e.g. 6 y = 6.00–6.99 y

**Table 4:** Age and sex specific percentiles of waist circumference/height ratio (WHTR) for Hong Kong Chinese children 6–18 y of age

			**Percentiles**								
			
**Sex**	**Age**	**n**	**3^rd^**	**5^th^**	**10^th^**	**25^th^**	**50^th^**	**75^th^**	**90^th^**	**95^th^**	**97^th^**
**Boys**	6	402	0.39	0.39	0.40	0.42	0.45	0.48	0.52	0.55	0.57
	7	520	0.38	0.39	0.40	0.41	0.44	0.47	0.51	0.54	0.57
	8	572	0.37	0.38	0.39	0.41	0.43	0.47	0.51	0.54	0.56
	9	629	0.37	0.37	0.38	0.40	0.43	0.46	0.50	0.53	0.55
	10	627	0.36	0.37	0.38	0.40	0.42	0.45	0.49	0.52	0.55
	11	644	0.36	0.36	0.37	0.39	0.42	0.45	0.49	0.52	0.54
	12	729	0.35	0.36	0.37	0.39	0.41	0.44	0.48	0.51	0.53
	13	657	0.35	0.35	0.36	0.38	0.40	0.44	0.47	0.50	0.53
	14	632	0.34	0.35	0.36	0.38	0.40	0.43	0.47	0.50	0.52
	15	558	0.34	0.35	0.35	0.37	0.40	0.43	0.47	0.50	0.52
	16	579	0.34	0.34	0.35	0.37	0.40	0.43	0.47	0.50	0.52
	17	553	0.34	0.34	0.35	0.37	0.39	0.43	0.46	0.50	0.52
	18	370	0.34	0.34	0.35	0.37	0.39	0.43	0.46	0.50	0.52
											
**Girls**	6	377	0.38	0.38	0.39	0.41	0.43	0.46	0.49	0.51	0.52
	7	479	0.37	0.38	0.39	0.40	0.42	0.45	0.48	0.50	0.52
	8	504	0.37	0.37	0.38	0.40	0.42	0.44	0.47	0.49	0.51
	9	590	0.36	0.37	0.38	0.39	0.41	0.44	0.47	0.49	0.50
	10	584	0.36	0.36	0.37	0.39	0.41	0.43	0.46	0.48	0.50
	11	599	0.35	0.36	0.37	0.38	0.40	0.43	0.46	0.48	0.50
	12	750	0.35	0.35	0.36	0.38	0.40	0.42	0.45	0.47	0.49
	13	637	0.35	0.35	0.36	0.37	0.39	0.42	0.45	0.47	0.49
	14	656	0.34	0.35	0.36	0.37	0.39	0.42	0.45	0.47	0.49
	15	600	0.34	0.35	0.36	0.37	0.39	0.42	0.45	0.47	0.49
	16	640	0.34	0.35	0.36	0.37	0.39	0.42	0.45	0.47	0.49
	17	568	0.34	0.35	0.36	0.37	0.39	0.42	0.45	0.47	0.49
	18	386	0.34	0.35	0.36	0.37	0.39	0.42	0.45	0.47	0.49

Age: completed age, e.g. 6 y = 6.00–6.99 y

**Figure 3:**
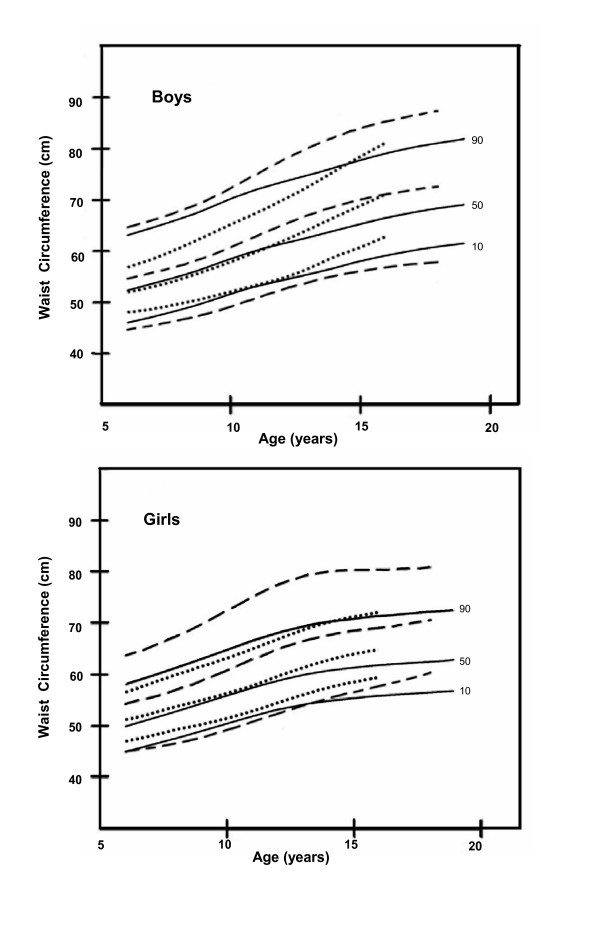
**Comparison of smoothed percentile curves of waist circumference for UK, Iran and Hong Kong children**. ______ Hong Kong (2005–2006), _ _ _ _ Iran(2003–2004), ............. UK(1978 & 1988).

Comparison of the 10^th^, 50^th ^and 90^th ^percentile curves of WC with the 1988 British and 2003/4 Iranian data showed that both Hong Kong boys and girls were closer to British children except that our 90^th ^percentile curve for boys was higher. Our 50^th ^and 90^th ^percentiles were lower than the Iranian curves (Figure 3). Comparison of WHTR data of Hong Kong boys and girls was made with the British data (the only dataset which provided WHTR for each year group) showed they are very similar with the mean differences ranging from only zero to 0.02 (Table 5).

**Table 5:** Mean (SD) of waist height ratio at yearly intervals in Hong Kong and British children

	Boys			Girls		
				
Age (y)	HK^a^	UK^b^	p-value	HK^a^	UK^c^	p-value
6	0.45(0.04)	0.45(0.03)	0.999	0.43(0.03)	0.45(0.03)	< 0.0001
7	0.44(0.04)	0.44(0.03)	0.999	0.43(0.04)	0.44(0.03)	< 0.0001
8	0.43(0.05)	0.43(0.03)	0.999	0.42(0.04)	0.43(0.03)	0.861
9	0.44(0.06)	0.43(0.04)	0.007	0.42(0.04)	0.42(0.03)	0.999
10	0.44(0.06)	0.43(0.04)	0.005	0.41(0.04)	0.41(0.04)	0.999
11	0.43(0.05)	0.43(0.04)	0.999	0.40(0.04)	0.41(0.04)	< 0.0001
12	0.42(0.05)	0.43(0.04)	0.001	0.40(0.04)	0.41(0.03)	< 0.0001
13	0.40(0.05)	0.42(0.04)	< 0.0001	0.40(0.04)	0.41(0.03)	< 0.0001
14	0.44(0.05)	0.42(0.04)	< 0.0001	0.40(0.04)	0.40(0.03)	0.999
15	0.40(0.05)	0.42(0.04)	< 0.0001	0.40(0.04)	0.40(0.03)	0.999
16	0.40(0.05)	0.42(0.04)	< 0.0001	0.40(0.04)	0.41(0.03)	< 0.0001

^a^Hong Kong data collected in 2005–2006, ^b^UK boys' data collected in 1977, and ^c ^UK girls' data collected in 1987

## Discussion

Sex and age-specific WC and WHTR percentiles, developed from data collected during 2005/6 on a large and representative population of 14,842 Chinese children aged six to 18 y old, are presented. These charts are based on WC measured midway between lowest rib and superior iliac crest. Comparisons of WC data between different studies need to be undertaken with caution since WC may be measured at different sites and currently there is no agreement on which site is optimal [34]. In children, WC has been measured at five different sites: (i) midway between lowest rib and superior iliac crest (as in the present study) [20,22,25,27]; (ii) at the umbilical level [19,21,28]; (iii) at the narrowest point of the torso [23]; (iv) at the level of the right upper iliac crest [24]; and (v) at the level of 2 cm above the umbilicus [35]. WC measures at these different sites in children have not been formally compared. WC measurements in adults taken at four different sites have been compared and shown to provide slightly different, albeit highly reproducible data, that each correlate significantly with total and central body fat [34]. If WC is to become an important public health assessment tool of central obesity in both adults and children, international agreement about measurement site is required.

In the study, we compared Hong Kong children's WC with data from Britain and Iran where the method of measuring WC was the same as our study. The Iranian children's 50^th ^and 90^th ^percentile curves were much higher than those from Hong Kong and Britain. A previous report has shown that Iranian children have a high prevalence of high blood triglyceride (20%) and low HDL-C (28%) [6]. In contrast, our unpublished data has shown much lower prevalence of these risk factors in Hong Kong children. The Iranian paediatric population may provide an important opportunity to further study the relation between central obesity and dyslipidaemia.

Our study challenges the proposal that WHTR is a relatively age-independent measure that could obviate the need for age and growth-related reference standards in children. Our data show that WHTR is not independent of age in children under 14 y old. Kahn and colleagues reported that WHTR appears to be independent of age and sex [36]. Ashwell and colleagues have proposed the use of an age-independent universal cut-off for WHTR of 0.5 [29]. This is despite their finding that the overall mean WHTR (with WC measured at the same site as in the present study) of 8135 British children aged 5 to 16 y decreased with age from 0.47 to 0.42 in boys and from 0.46 to 0.41 in girls [31,37]. WHTR measures obtained in our study were slightly less than the 1988 British study. Our WHTR decreased with age up to 14 y and then remained almost constant (Table 1, Figure 2). WHTR has now been correlated with CV risk markers in children in at least six studies [6,28,30,35,36,38] and different WHTR cut-offs for predicting high CV risks in children at different ages were reported in one of them [6]. Ashwell's proposed 0.5 cut-off point for predicting CVS risk has yet to be tested by direct correlation with CV risk markers in children. Notably, few of our older children would be categorized as being at CV risk if this single 0.5 WHTR cut-off were used (Figure 2). This would conflict with our previous findings that a WC cut-off at the 85^th ^percentile for children under the age of 12 y and at the 76^th ^percentile for those aged 13 to 18 y would predict CV risk [11,39]. For a WHTR cut-off of 0.5 to become a universal predictor of CV risk in children, further direct confirmatory evidence from comparative correlations with CV risk factors in children of different age and ethnic origins will be required.

BMI is the most widely used index to define children's weight status. Our finding that WC correlates considerably more closely than does WHTR with BMI in children is unsurprising given that WC and BMI increase, whereas WHTR decreases, with age. CV risk factors found in our previous study of around 2500 children drawn from the same population correlated in descending order of strength with WC, BMI and WHTR [11]. Others have reported that WC [28,39-41] and WHTR [28,30,36] correlate more strongly than does BMI with CV risk factors in children. No difference in the ability of WHTR and BMI-for-age to predict CV risk was found in one study [38]. These inconsistencies may reflect different methods of analysis, different ethnic populations and the relatively small differences between the related parameters.

A limitation of our study is that 7% of the primary and 10% of the secondary school students declined to participate. Reasons for non-participation were not recorded so it is not possible to determine whether non-participation resulted in any systematic bias.

## Conclusion

Reference values and percentile curves for WC and WHTR of Chinese children and adolescents are provided in the study. Both WC and WHTR were age dependent. As a public health tool, WHTR does not on present evidence offer major advantages over WC for children. WC, measured at an agreed site and interpreted against appropriate established reference standards, remains the simplest clinical measure of central obesity for the prediction of CV risk in children. For standards based on WC and WC-derived parameters to be of general public health and research use for international comparisons, it will be important that a single defined site be agreed at which to measure WC.

## Competing interests

The authors declare that they have no competing interests.

## Authors' contributions

RYTS, EASN and AML prepared the proposal and supervised the study. HKS coordinated the study, assisted in the supervision of data collection, and took part in the statistical work. KCC took active part in the data analysing. JATY and CWLK helped in data collection and develop the protocol. PCN and TFF made substantial contributions to the conception of the study and revising the manuscript. All authors read and approved the final manuscript.

## Pre-publication history

The pre-publication history for this paper can be accessed here:


